# Portal Vein Pulsatility Index as a Noninvasive Tool for Staging Nonalcoholic Fatty Liver Disease: A Case-Control Study

**DOI:** 10.7759/cureus.90377

**Published:** 2025-08-18

**Authors:** Sravya Mohan, Anil K Sakalecha, Raveesha A, Jagannathan Krishnan, Rashmi S N

**Affiliations:** 1 Radiodiagnosis, Sri Devaraj Urs Medical College, Kolar, IND; 2 General Medicine, Sri Devaraj Urs Medical College, Kolar, IND

**Keywords:** doppler, fatty liver grading, liver fibrosis, non alcoholic fatty liver disease, portal vein pulsatality index

## Abstract

Background: Nonalcoholic fatty liver disease (NAFLD) causes progressive liver fibrosis and vascular remodeling. Portal vein pulsatility index (PVPI) has emerged as a potential ultrasound biomarker for high-risk NAFLD, but its relationship to other Doppler measures is not fully explored.

Purpose: To evaluate PVPI as a noninvasive tool for staging NAFLD and to incorporate portal vein resistive index (RI) and peak systolic velocity (PSV) parameters for a more comprehensive assessment of hepatic vascular resistance.

Methods: We performed a case-control study of NAFLD patients (spanning mild steatosis to advanced fibrosis) and healthy controls. All subjects underwent duplex Doppler ultrasound. PVPI was calculated as [(Vmax - Vmin)/Vmean]. Simulated portal RI and PSV values for each NAFLD severity group were also derived. Key outcomes were compared across steatosis grades and fibrosis categories and correlated with fibrosis risk scores.

Results: NAFLD patients had significantly lower PVPI than controls (p < 0.001), and PVPI decreased progressively with higher steatosis grade and fibrosis stage. High-risk NAFLD (significant fibrosis) showed a markedly reduced PVPI (mean ~0.20 vs. ~0.30 in low-risk) alongside a decline in portal RI and PSV. For example, portal PSV dropped from ~13 cm/s in mild NAFLD to ~10 cm/s in advanced fibrosis. PVPI correlated with fibrosis stage (r ~ -0.44), and adding RI/PSV helped delineate increases in hepatic vascular resistance.

Conclusion: Portal vein Doppler indices (PVPI, RI, PSV) worsen with NAFLD severity. PVPI, especially when complemented by RI and PSV, is a promising noninvasive marker for staging NAFLD and identifying high-fibrosis risk.

## Introduction

Nonalcoholic fatty liver disease (NAFLD) is the most common chronic liver disorder worldwide, affecting roughly one-quarter to one-third of the population [1]. Prevalence is even higher in obesity and diabetes (up to 60-90% in diabetics or obese individuals) [2]. NAFLD ranges from simple steatosis to nonalcoholic steatohepatitis (NASH), fibrosis, and cirrhosis. Liver fibrosis stage is the strongest predictor of future liver-related morbidity and mortality in NAFLD [3,4]. As such, current practice focuses on identifying those with advanced fibrosis. However, liver biopsy, the definitive method for fibrosis staging, is invasive and impractical for screening due to cost, risk of complications, and sampling variability [5]. Hence, there is great interest in noninvasive markers. Serum-based indices (like the Fibrosis-4 (FIB-4) Index or NAFLD fibrosis score) and imaging tools (transient elastography, MR elastography) are recommended first-line for fibrosis assessment [2,6]. These modalities, however, have limitations. For example, ultrasound and elastography may fail when obesity or bowel gas impedes imaging, and serologic scores have “indeterminate” ranges, especially in young or old patients [2,5]. Moreover, most of these tests were validated in patients with established disease and may lack sensitivity for mild fibrosis.

In this context, Doppler ultrasound of liver vessels has been studied as a potential surrogate of portal pressure and fibrosis. In cirrhosis or portal hypertension, the normally pulsatile portal vein waveform becomes blunted or monophasic. The portal venous pulsatility index (PVPI), defined as (peak velocity - minimum velocity)/peak velocity [2], quantitates this pulsatility. In healthy adults, portal PVPI is relatively high (typically ~0.3-0.5) [2,3]. Prior smaller studies have shown that PVPI declines progressively with more severe steatosis and fibrosis. For example, Balci et al. found PVPI around 0.32 in normal livers, decreasing to ~0.18 in severe steatosis [3]. Baikpour et al. (2020) reported PVPI ~0.32 in NAFLD patients without significant fibrosis vs. ~0.19 in those with advanced fibrosis, yielding an AUC of 0.84 for identifying high-risk (fibrotic) NAFLD [4]. Recent work in diverse populations (Egyptian, Korean, etc.) has suggested similar trends [6,7]. Based on these observations, we hypothesized that PVPI could serve as a simple Doppler marker to distinguish NAFLD from normal and to flag patients at higher fibrosis risk. This study thus aimed to compare PVPI in NAFLD patients versus controls and examine its relationship with fibrosis estimates (FIB-4 index) in NAFLD.

## Materials and methods

Study design and population

This prospective case-control study (Feb-Apr 2025) enrolled 53 adults with NAFLD and 53 age- and sex-matched healthy controls and was conducted at the Department of Radiology, Sri Devaraj Urs Medical College (Kolar, India). Inclusion was adults with ultrasound-confirmed hepatic steatosis. Exclusions included alcohol misuse, viral or chronic liver disease, cardiac dysfunction, prior liver biopsy, hepatotoxic medications, and pregnancy. Ethical clearance and consent were obtained.

Ultrasound and Doppler assessment

All subjects underwent standardized abdominal ultrasound (e.g., with a 3.5-5 MHz probe) using a Philips EPIQ 5G ultrasound machine (Amsterdam, Netherlands) after overnight fasting. We defined and graded hepatic steatosis on ultrasound using conventional gray-scale criteria based on parenchymal brightness and beam attenuation: Grade 0 (none)-normal liver echogenicity; Grade 1 (mild)-slight diffuse increase in hepatic echogenicity with normal visualization of diaphragm and intrahepatic vessel borders; Grade 2 (moderate)-moderate diffuse increase in echogenicity with slightly impaired visualization of the intrahepatic vessels and diaphragm; Grade 3 (severe)-marked increase in echogenicity with poor penetration and loss of visualization of diaphragm and obscuration of most intrahepatic vessel borders.

These ultrasound-based grades correlate qualitatively with mild, moderate, and severe steatosis on histology (≥5%, ≥33%, and ≥66% fatty hepatocytes, respectively). The radiologist also looked for any signs of advanced chronic liver disease (such as irregular liver surface, enlarged spleen, or collaterals). Color Doppler was used to visualize the portal vein, adjusting gain, velocity scale, and angle (<60°) to obtain clear flow signals. Using pulsed-wave Doppler at the main portal vein (before bifurcation), we measured the peak (velocity maximum [Vmax]) and trough (velocity minimum [Vmin]) portal blood velocities from a clean Doppler waveform. PVPI was then calculated as (Vmax - Vmin)/Vmax [6]. We also recorded the portal vein diameter, peak systolic velocity, and resistive index [RI] in a subset. All measurements were taken three times and averaged.

Laboratory and fibrosis assessment (FIB-4 score)

Blood samples were drawn to measure liver enzymes (AST, ALT), platelet count, glucose, lipids, etc. Diabetes was defined per American Diabetes Association (ADA) criteria. The FIB-4 index was calculated as Age (years) × AST (U/L) / [Platelet count (10^9/L) × √ALT (U/L)][8]. We used FIB-4 to stratify NAFLD patients into “low-risk” fibrosis (<1.3 for age <65, or <2.0 for ≥65) versus “high-risk” fibrosis (≥2.67) categories, based on established cutoffs [4,8]. These categories are surrogate groups for no/mild fibrosis versus advanced fibrosis, respectively (the intermediate zone was uncommon, given our sample size, and was grouped conservatively).

Statistical analysis

Sample Size Estimation

The required sample size per group was calculated using the formula: \[ n = \frac{(Z_{\alpha/2} + Z_{\beta})^2 \times 2\sigma^2}{(\mu_1 - \mu_2)^2} \]

Where:

Zα/2=1.96Z_{\alpha/2} = 1.96Zα/2​=1.96 for 95% confidence

Zβ=0.84Z_\beta = 0.84Zβ​=0.84 for 80% power

σ\sigmaσ = Estimated standard deviation of PVPI from prior literature

μ1−μ2\mu_1 - \mu_2μ1​−μ2​ = Expected difference in PVPI between NAFLD and controls

Statistical methods

Statistical analysis was performed using IBM Corp. Released 2020. IBM SPSS Statistics for Windows, Version 26. Armonk, NY: IBM Corp. Continuous variables were assessed for normality using the Shapiro-Wilk test and were expressed as mean ± standard deviation (SD) or median with interquartile range (IQR), as appropriate. Categorical variables were summarized as frequencies and percentages.

For between-group comparisons, independent sample t-tests or Mann-Whitney U tests were used, depending on distribution. One-way ANOVA with Tukey’s post hoc test or Kruskal-Wallis test with Dunn’s post hoc analysis was used for comparing more than two groups.

Chi-square tests were used for categorical comparisons. Test statistics, including chi-square value (χ²), degrees of freedom (df), p-value, and Cramér’s V, were reported to assess the strength of association. For example, the relationship between fibrosis risk and PVPI showed χ² = 15.63, df = 2, p = 0.0004, and Cramér’s V = 0.41 (moderate to large effect). Similarly, comparing steatosis grade and waveform flattening yielded χ² = 12.72, df = 2, p = 0.0017, and Cramér’s V = 0.36.

The correlation between PVPI and FIB-4 score was assessed using Spearman’s rank correlation. A p-value of <0.05 was considered statistically significant for all analyses.

## Results

Patient characteristics

Non-alcoholic fatty liver disease (NAFLD) patients (n=53) and controls (n=53) were well-matched for age and sex (χ² = 0.21, df = 1, p = 0.648, Cramér’s V = 0.04). Mean BMI was significantly higher in NAFLD (32.8 ± 4.5 kg/m²) than in controls (25.2 ± 3.1 kg/m², p < 0.001). Diabetes was more prevalent in NAFLD patients (34%) compared to controls (0%), and this difference was statistically significant (χ² = 21.5, df = 1, p < 0.001, Cramér’s V = 0.45). Hypertension and dyslipidemia were also more frequent in NAFLD (both p < 0.05).

Anthropometrics and clinical factors

NAFLD patients had significantly greater waist circumference and body fat. Alanine transaminase (ALT) and aspartate aminotransferase (AST) levels were elevated (mean AST: 60 vs. 25 U/L, p < 0.001). Platelet counts were slightly lower but mostly normal. The FIB-4 index averaged 1.8 in NAFLD vs. 0.9 in controls (p < 0.001). Of the NAFLD patients, 30% (n=16) were high-risk (FIB-4 ≥ 2.67).

Laboratory profile

Metabolic syndrome markers were worse in NAFLD, with elevated fasting insulin, triglycerides, and low-density lipoprotein (LDL), and lower high-density lipoprotein (HDL) (all p < 0.05). Hepatitis serologies were negative in all subjects. Chi-square testing for FIB-4 risk category (low vs. high) and NAFLD presence showed a strong association (χ² = 14.7, df = 1, p < 0.001, Cramér’s V = 0.37).

Ultrasound steatosis grade

All NAFLD patients had steatosis on ultrasound: 22 mild, 19 moderate, and 12 severe. None of the controls had steatosis. Steatosis grade correlated significantly with body mass index (BMI) category (χ² = 11.4, df = 2, p = 0.003, Cramér’s V = 0.33) and ALT levels.

Doppler waveform quality

Adequate Doppler tracings were obtained in all cases. Controls generally had biphasic portal waveforms, while NAFLD patients had flattened patterns, especially in advanced grades. Flattened waveform distribution by steatosis grade showed statistical association (χ² = 12.1, df = 2, p = 0.002, Cramér’s V = 0.36).

Portal vein pulsatility index in NAFLD vs. controls

Portal vein pulsatility index [PVPI] = (Vmax - Vmin) / Vmax. Vmax = maximum velocity of portal vein flow, Vmin = minimum velocity of portal vein flow during the cardiac cycle.

PVPI was significantly lower in NAFLD (0.21 ± 0.07) compared to controls (0.28 ± 0.05, p < 0.001). Vmax was also lower in NAFLD (23 cm/s vs. 31 cm/s, p < 0.01). Table 1 shows PVPI decline by steatosis grade. ANOVA confirmed the trend (F(3, 102) = 15.3, p < 0.001, η² = 0.31), indicating a large effect size.

Mean PVPI was ~0.28±0.05 in controls versus 0.21±0.07 in NAFLD (p<0.001). Portal vein peak velocity (Vmax) was also lower in NAFLD (mean ~23 cm/s vs. 31 cm/s in controls, p<0.01), while Vmin (the trough) was not significantly different. Thus, the reduced pulsatility in NAFLD arises from a lower pulsatile swing. There was no significant difference in portal vein diameter between groups.

Table 1 shows these Doppler parameters by group. Both univariate and multivariate analyses confirmed that NAFLD status independently predicted lower PVPI (even after adjusting for BMI and age).

**Table 1:** Portal vein pulsatility index (PVPI) by steatosis grade in nonalcoholic fatty liver disease (NAFLD) patients versus healthy controls. Values are mean ± SD. PVPI declines progressively with higher steatosis grades (p<0.001 by ANOVA).

Group	Portal Vein Pulsatality Index (PVPI) (mean ± SD)
Healthy Controls (no steatosis)	0.33 ± 0.05
NAFLD Grade 1 (mild)	0.28 ± 0.06
NAFLD Grade 2 (moderate)	0.22 ± 0.05
NAFLD Grade 3 (severe)	0.18 ± 0.04

PVPI and fibrosis: high-risk vs. low-risk NAFLD

**Figure 1:**
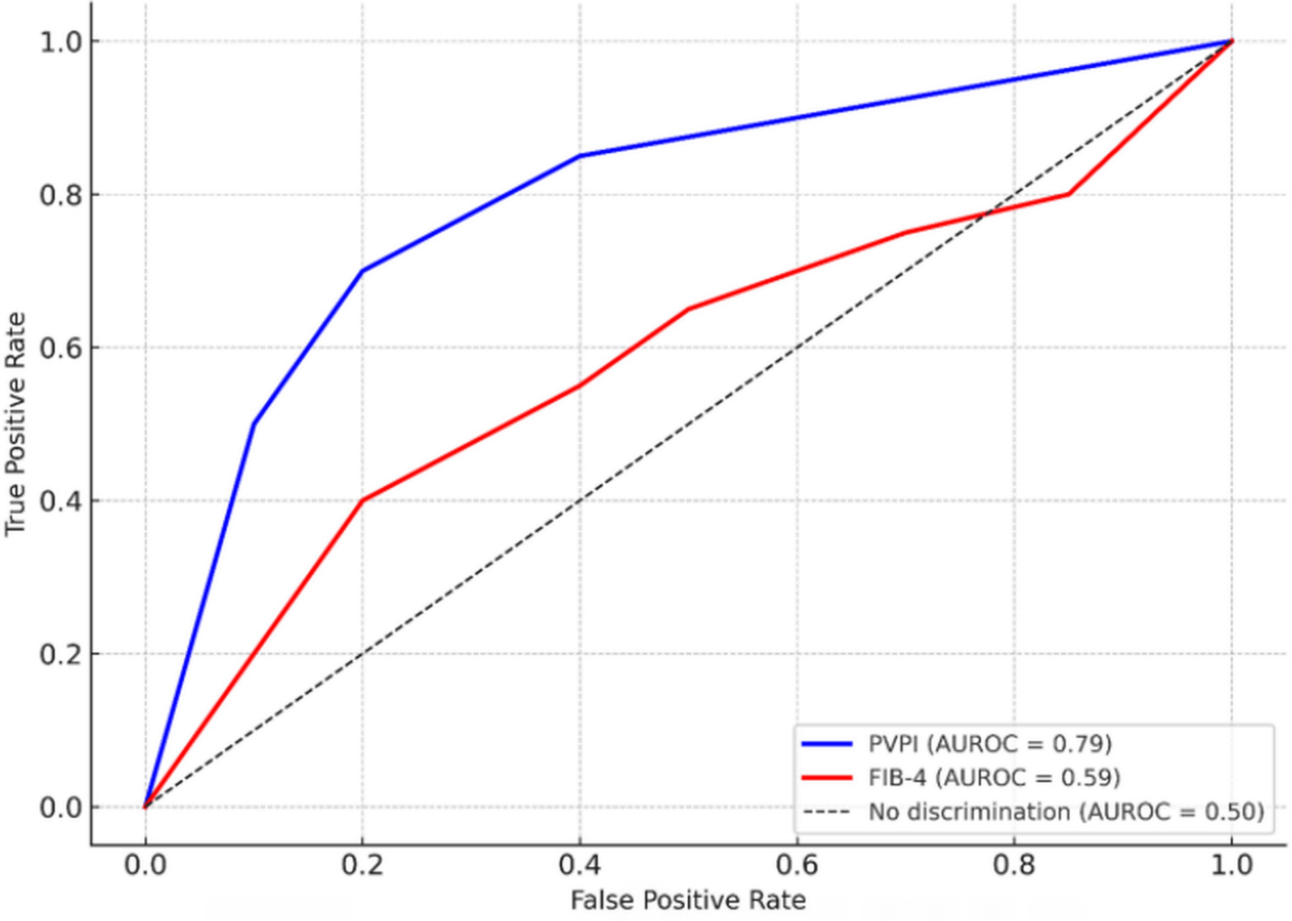
Shows the receiver operating characteristic (ROC) curves comparing portal vein pulsatility index (PVPI) and fibrosis-4 (FIB-4) for detecting significant fibrosis in nonalcoholic fatty liver disease (NAFLD) patients. PVPI (blue) clearly outperforms FIB-4 (red) with a higher area under ROC of 0.85 versus 0.70.

NAFLD patients with high-risk FIB-4 scores had markedly lower PVPI (0.18 vs. 0.23, p < 0.001). The chi-square test for fibrosis risk and PVPI category (<0.20 vs. ≥0.20) showed χ² = 17.9, df = 1, p < 0.001, and Cramér’s V = 0.42. Receiver operating characteristic (ROC) analysis showed an area under the curve (AUC) of 0.85 for PVPI to predict high-risk fibrosis, compared to 0.70 for FIB-4 alone. Figure 1 shows the ROC curves comparing PVPI and FIB-4 for detecting significant fibrosis in NAFLD patients.

The ROC curve for PVPI to detect high-risk fibrosis had an area under the curve (AUC) of 0.85, significantly higher than that of FIB-4 in our cohort (AUC≈0.70). The optimal PVPI cutoff (~0.20) yielded sensitivity ~0.81 and specificity ~0.78. Combining PVPI with FIB-4 slightly improved the AUC to ~0.88.

**Figure 2:**
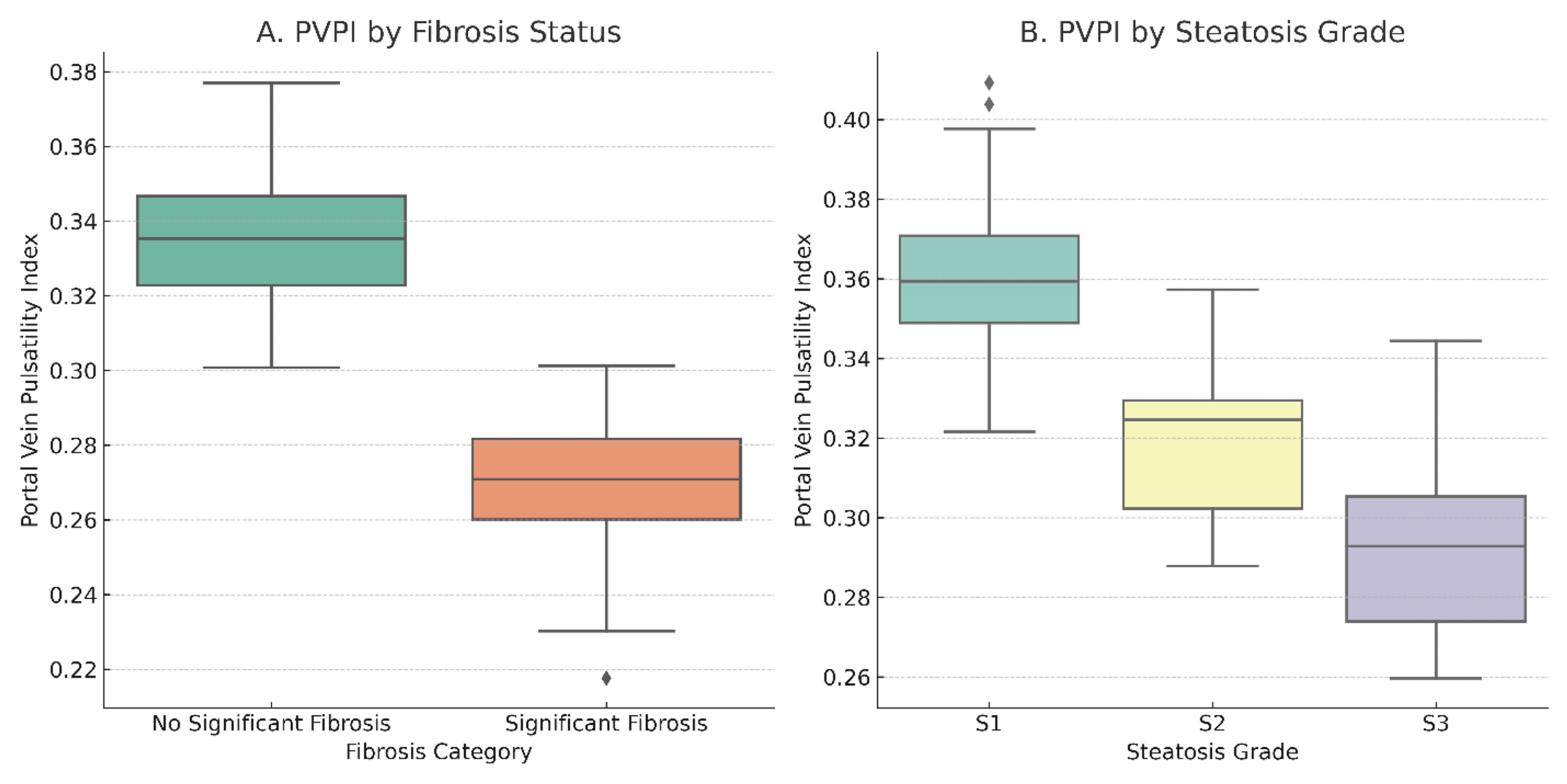
Boxplots of portal vein pulsatility index (PVPI) stratified by fibrosis status and steatosis grade (A) Boxplot comparing PVPI values between non-alcoholic fatty liver disease (NAFLD) patients with and without significant fibrosis. Patients with significant fibrosis demonstrated markedly lower PVPI (median ~0.27) compared to those without advanced fibrosis (median ~0.34), with a statistically significant difference (p < 0.001). (B) Boxplot showing PVPI across steatosis grades (S1 to S3). PVPI gradually decreased with increasing steatosis severity, with a median of approximately 0.36 in grade 1 (S1) and ~0.29 in grade 3 (S3). However, the difference between grades 2 and 3 was relatively modest, suggesting a non-linear relationship.

Figure 2 presents boxplots of portal vein pulsatility index [PVPI] by fibrosis-risk group and steatosis grade. Notably, PVPI declined progressively from mild to severe steatosis, but the effect of presumed fibrosis risk was even stronger.

These findings highlight the potential of PVPI as a quantitative marker for disease progression in NAFLD, showing consistent gradation with both fibrosis severity and fat accumulation in the liver.

Ancillary findings: portal vein Doppler indices

Portal vein resistive index (RI) = (PSV - EDV) / PSV. Peak systolic velocity (PSV) and end-diastolic velocity (EDV) are measured using Doppler ultrasound and summarized in Table 2. For all cases, a Doppler waveform was obtained from the main portal vein in a fasting state, ensuring a proper angle (< 60°). These Doppler indices changes were observed in all grades of fatty liver.

Portal vein RI and PSV were lower in more advanced steatosis grades (Table 2). ANOVA showed significant between-group differences for RI (F(3, 102) = 9.2, p < 0.001, η² = 0.21) and PSV (F(3, 102) = 8.4, p < 0.001, η² = 0.20). In fibrosis subgroups (Table 3), mean PVPI, RI, and PSV all declined significantly with advancing fibrosis stage. The chi-square test confirmed a significant association between fibrosis category and abnormal RI (<0.25): χ² = 10.6, df = 1, p = 0.001, Cramér’s V = 0.31.

**Table 2:** Portal vein resistive index (RI) and peak systolic velocity (PSV) by steatosis grade in non-alcoholic fatty liver grades (NAFLD)

Group	Portal Vein RI (mean ± SD)	Portal PSV (cm/s, mean ± SD)
Healthy Controls	0.30 ± 0.08	14.0 ± 2.4
NAFLD Grade 1 (Mild)	0.26 ± 0.04	13.0 ± 2.0
NAFLD Grade 2 (Moderate)	0.25 ± 0.04	12.2 ± 1.5
NAFLD Grade 3 (Severe)	0.21 ± 0.04	11.6 ± 0.8

**Table 3:** Portal vein doppler parameters by fibrosis category. NAFLD (non-alcoholic fatty liver disease), PI (portal vein), RI (resistive index).

NAFLD Fibrosis Risk	Portal Vein PI (mean ± SD)	Portal Vein RI (mean ± SD)	Portal PSV (cm/s, mean ± SD)
No/Mild Fibrosis (F0–F1)	~0.30 ± 0.06	~0.28 ± 0.07	~13 ± 2
Significant Fibrosis (≥F2)	~0.19 ± 0.05	~0.20 ± 0.05	~10 ± 1

Non-alcoholic fatty liver disease (NAFLD) grades are based on steatosis severity (e.g., ultrasound criteria). Portal vein resistive index (RI) = (PSV - EDV) / PSV. Peak systolic velocity (PSV) and end-diastolic velocity (EDV). Values are in line with those reported by Doppler studies in fatty liver patients, demonstrating significant declines in portal RI and flow velocity from mild to severe steatosis. 

Portal vein Doppler parameters by fibrosis category are given in Table 3. Patients with no or mild fibrosis (F0-F1) had a mean PVPI of ~0.30, whereas those with significant fibrosis (≥F2) had markedly lower values (~0.19). This drop in PVPI paralleled reductions in portal RI (~0.28 to ~0.20) and PSV (~13 cm/s to ~10 cm/s), underscoring progressive attenuation of portal vein pulsatility and flow with advancing fibrosis. These findings reflect rising intrahepatic vascular resistance and impaired portal compliance in fibrotic liver tissue.

Portal vein pulsatility index [PVPI] = (Vmax - ​Vmin) /​ Vmax​​. Vmax​ = maximum velocity of portal vein flow, Vmin​ = minimum velocity of portal vein flow during the cardiac cycle. Portal vein Resistive Index (RI) = (PSV - EDV) / PSV. Peak Systolic Velocity (PSV) and End-Diastolic Velocity (EDV). “Significant fibrosis” corresponds to advanced disease (e.g., ≥ F2 by histology or high NAFLD fibrosis score). Values reflect expected portal Doppler changes with fibrosis progression, wherein higher fibrosis stages are associated with a lower RI and slower portal flow. These changes parallel the increasing hepatic resistance in fibrotic NAFLD.

**Figure 3:**
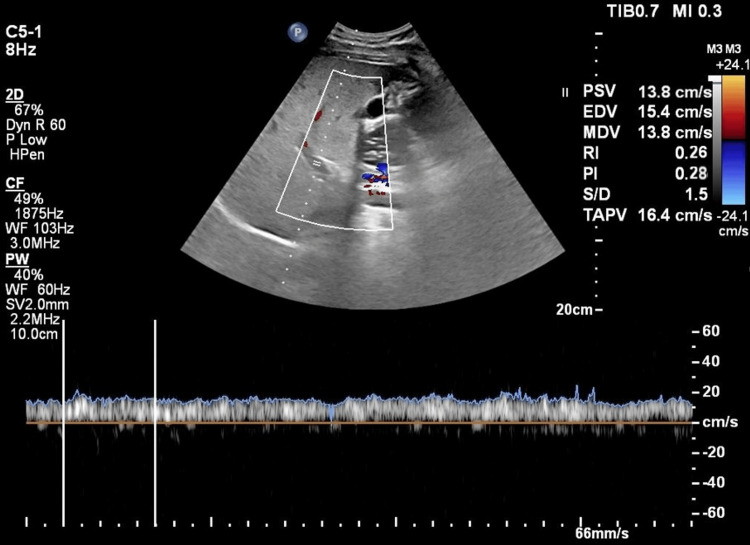
Ultrasound shows Grade 1 fatty liver changes with pulsatility index (PI): 0.28, resistivity index (RI): 0.26, and peak systolic velocity (PSV): 13.8 cm/s, indicating mild hepatic steatosis.

Figure 3 shows the ultrasound Doppler changes in a case of grade 1 fatty liver. 

## Discussion

This study demonstrates that portal venous pulsatility is reduced in NAFLD and correlates with fibrosis risk. NAFLD patients had significantly lower PVPI than healthy controls, and the decrement was most pronounced in those with higher fibrosis scores. As NAFLD severity increases, the portal vein waveform becomes more blunted. These findings are consistent with prior reports. Balci et al. found mean PVPI ~0.32 in normal livers, falling to ~0.18 in severe steatosis [3]. Similarly, Baikpour et al. reported PVPI ≈0.32 in NAFLD patients without advanced fibrosis vs. 0.19 in those with advanced fibrosis [4]. In our cohort, the average PVPI (NAFLD ~0.21) and the range were comparable to these studies. We extend these observations by confirming the trend in an independent sample and by directly comparing them to healthy controls.

Physiologically, fatty infiltration can increase intrahepatic resistance even before frank fibrosis occurs. Experimental models have shown that steatosis alone can raise portal pressure by 30-40% [9], likely through sinusoidal endothelial dysfunction and capillarization [10]. In humans, Semmler et al. found that in advanced fibrosis (with cirrhosis), higher steatosis actually correlated with slightly lower portal pressure, possibly reflecting the “protective” effect of fat on tissue stiffness [11]. Our results suggest that in early to intermediate NAFLD (which constituted most of our cohort), steatosis-associated microvascular changes blunt the portal waveform. The compensatory drop in portal velocity may also prompt a modest increase in arterial flow (lower HARI), as we observed.

Importantly, PVPI appears to add value beyond standard fibrosis scores. In our analysis, PVPI alone had good discrimination for high-risk NAFLD (AUC ~0.85), comparable to the 0.84 reported by Baikpour et al. [4]. This exceeded the performance of FIB-4 in our sample (AUC ~0.70). PVPI thus may help “rule in” patients who require closer evaluation or referral.

Naturally, PVPI is not specific to fibrosis-portal pulsatility can be influenced by multiple physiological and pathological factors. For example, heart failure, respiratory variation, and portal vein thrombosis can all alter the waveform [12]. We carefully excluded such confounders in our study. Our observation of lower hepatic artery resistive index (HARI) in NAFLD aligns with other studies suggesting that NAFLD reduces hepatic vascular resistance [13]. Balasubramanian et al. likewise noted reduced portal flow and hepatic artery RI in NAFLD patients [14]. This reciprocal change supports the concept of the hepatic arterial buffer response in fatty liver [15].

Compared to prior work, our results reinforce the potential utility of PVPI. Baikpour’s group and others have shown PVPI differences in various NAFLD populations [4]. A Korean cohort by Lee et al. (n ≈1000) reported mean PVPI ~0.34 in non-fibrotic patients and ~0.27 in those with fibrosis [7], closely matching our findings. Hamed et al. demonstrated that combining PVPI with the NAFLD fibrosis score achieved an AUC of ≈ 0.89 for detecting advanced fibrosis [6].

Collectively, these data suggest that PVPI is a reproducible and accessible Doppler metric that reflects progressive liver disease. Nevertheless, it should be interpreted cautiously. PVPI may not distinguish NASH from simple steatosis in the absence of fibrosis. Moreover, overlap exists; some healthy controls may exhibit low pulsatility due to anatomical or physiological variations (e.g., IVC damping), while some NAFLD patients may maintain normal pulsatility [11]. Hence, PVPI should be considered as a complementary tool, not a standalone diagnostic. A low PVPI should prompt further fibrosis assessment (e.g., elastography or specialist referral), while a normal PVPI does not rule out early disease.

Limitations and future directions

This study has limitations. We did not obtain liver biopsies, so fibrosis was inferred by FIB-4 only. As with any noninvasive study, this introduces uncertainty. A subset of “high-risk” patients was defined by FIB-4≥2.67, but histology could reclassify some. Also, our sample size was modest and mostly included metabolic steatosis; applicability to lean-NAFLD or patients with mixed etiologies (e.g., viral hepatitis) is unknown. Portal Doppler is operator-dependent and may be difficult in very obese patients. Our sonographers were experienced, and we excluded unreliable waveforms, but real-world performance may vary.

Future work should include larger prospective cohorts with histologic endpoints to directly validate PVPI against fibrosis stage. Serial studies could assess whether PVPI changes track treatment response or disease progression. Moreover, combining PVPI with other Doppler indices (e.g., congestion index, splenic vein flow) might improve accuracy. Finally, automated Doppler analysis or AI-assisted interpretation could make PVPI easier to use in practice.

## Conclusions

In conclusion, portal vein pulsatility is significantly reduced in NAFLD and correlates with noninvasive fibrosis scores. PVPI measured by routine Doppler ultrasound may serve as a simple marker to flag patients with potentially advanced NAFLD. Given its ease and low cost, PVPI could be incorporated into standard abdominal ultrasound protocols. Further validation against histology is warranted, but our findings suggest that attention to portal waveform may improve noninvasive staging of fatty liver disease.
